# Magnetite-based adsorbents for sequestration of radionuclides: a review

**DOI:** 10.1039/c7ra12299c

**Published:** 2018-01-11

**Authors:** Syed M. Husnain, Wooyong Um, Woojin-Lee, Yoon-Seok Chang

**Affiliations:** School of Environmental Science and Engineering, Pohang University of Science and Technology (POSTECH) Pohang 790-784 Republic of Korea; Division of Advanced Nuclear Engineering, POSTECH Republic of Korea; Chemistry Division, Directorate of Science, Pakistan Institute of Nuclear Science and Technology (PINSTECH) P.O. Nilore Islamabad 45650 Pakistan; Department of Civil Engineering, Nazarbayev University Astana 010000 Republic of Kazakhstan

## Abstract

As a result of extensive research efforts by several research groups, magnetite-based materials have gained enormous attention in diverse fields including biomedicine, catalysis, energy and data storage devices, magnetic resonance imaging, and environmental remediation. Owing to their low production cost, ease of modification, biocompatibility, and superparamagnetism, the use of these materials for the abatement of environmental toxicants has been increasing continuously. Here we focus on the recent advances in the use of magnetite-based adsorbents for removal of radionuclides (such as ^137^Cs(i), ^155^Eu(iii), ^90^Sr(ii), ^238^U(vi), *etc.*) from diverse aqueous phases. This review summarizes the preparation and surface modification of magnetite-based adsorbents, their physicochemical properties, adsorption behavior and mechanism, and diverse conventional and recent environmental technological options for the treatment of water contaminated with radionuclides. In addition, case studies for the removal of radionuclides from actual contaminated sites are discussed, and finally the optimization of magnetite-based remedial solutions is presented for practical application.

## Abstract 2

As a result of extensive research efforts by several research groups, magnetite-based materials have gained enormous attention in diverse fields.

## Graphical abstract

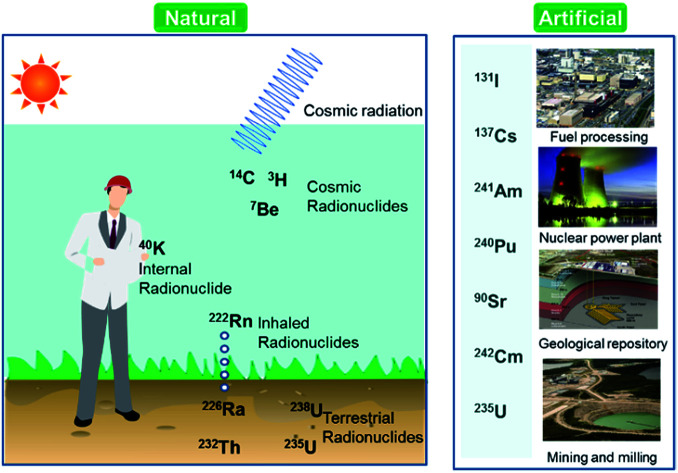

## 1. Introduction

**Fig. 1:**
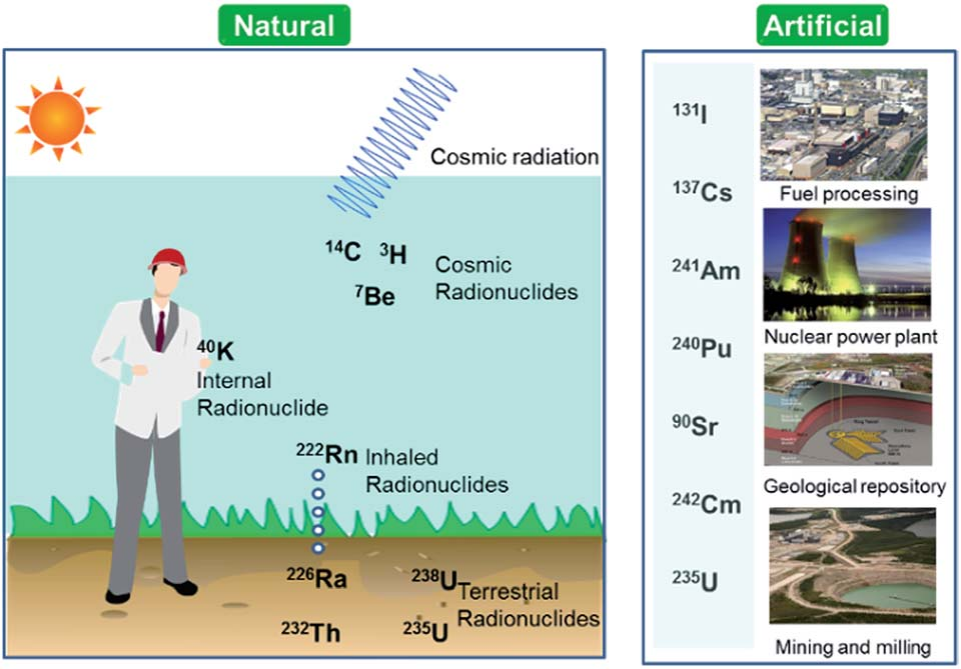
Classification of radionuclides in the environment.

Over the last few decades, there has been an increasing global concern over public health issues due to environmental pollution caused by radionuclides,^1,2^ heavy metals,^3^ and pesticides.^4^ Radionuclides are chemical elements emitting either α-, β-, or γ-rays, or neutrons. They can be classified on the basis of their origin such as naturally occurring and anthropogenic (Fig. 1). Among the naturally occurring radionuclides, there are further three types including primordial (^238^U, ^235^U, ^232^Th, ^4^ K), secondary (^210^Pb, ^26^Al, ^36^Cl, ^54^Mn *etc.*), and cosmogenic radionuclides (^3^H, ^14^C, ^7^Be *etc.*). Anthropogenic radionuclides include isotopes of ^239^Pu, ^129^I, ^137^Cs, ^99^Tc, and ^241^Am present in radioactive waste.^5^ Radioactive waste generated by nuclear facilities operation and weapon production can be categorized on the basis of radioactivity level such as low, intermediate, and high level waste. The major contaminant sources from which the radionuclides originate are nuclear power plants, nuclear weapon production and testing sites, commercial nuclear fuel processing units, and release from failed geological repositories.^2^

Radionuclides can reside in ambient air, soil, and water after their emission from sources, which is why a few radionuclides have been detected in soil, sediment, air, and aquatic environments. Past atmospheric weapons testing and some emissions from active nuclear power plants and irradiated fuel reprocessing activities can transfer radionuclides to the ground *via* rain water.^6^ They could be involved in complex reactions with soil organic matter.^7^ Their presence in upper layer of soil and sediment are two main sources for their introduction to the food chain.^8^ Some of anthropogenic and naturally occurring radionuclides such as ^222^Rn, ^226^Ra, ^228^Ra, ^238^U, ^234^U, and ^232^Th are commonly found in groundwater around the world.^9^ Exposure to the water contaminated with radionuclides may have more detrimental health concerns because of chemical as well as radiological toxicities. These radionuclides cause diseases such as neurological disorders, birth defects, infertility, and various types of cancers in different organs like lungs, thyroid, colon esophagus, breast, and ovary.^10–12^ Owing to the dual toxic nature, the U.S. Environmental Protection Agency (EPA) recommended maximum contaminant levels (MCLs) for radionuclides in water; specifically, 15 pCi L^−1^ (0.55 Bq) for alpha emitters, 4 mrem per year (40 μSv per year) for beta and photon emitters, 5 pCi L^−1^ (0.18 Bq L^−1^) for radium (^226^Ra/^228^Ra), and 30 μg L^−1^ (0.74 Bq L^−1^) for uranium have been established as drinking water standards.^13^

Monitoring and subsequent removal of these radionuclides from the contaminated water is one of the major environmental remediation interests today. Effluents discharged during the operation of nuclear facilities are regularly monitored to ensure the radioactivity level sufficiently lower than permissible limit. To treat the toxic radioactive contaminants, many conventional techniques including precipitation, reverse osmosis (RO), ion exchange (IE), electrodialysis, solvent extraction, and evaporation were developed and applied.^14–16^ Among these; precipitation, IE, and RO are widely used for *ex situ* treatment of groundwater contaminated with radionuclides. Precipitation is relatively simple, reliable, and cost effective to convert the majority of the soluble radionuclides into insoluble hydroxides, sulfides, or carbonates minerals. RO separates dissolved solids by passing the contaminated water through semipermeable membrane. Although the conventional technologies have made significant contributions to the environmental protection of human society in the last century, the continuous and ever-increasing demands for pure water is gradually pushing the conventional technologies to their limits and likely unable to meet strict regulations of the U.S EPA and the World Health Organization (WHO) standards.

**Fig. 2:**
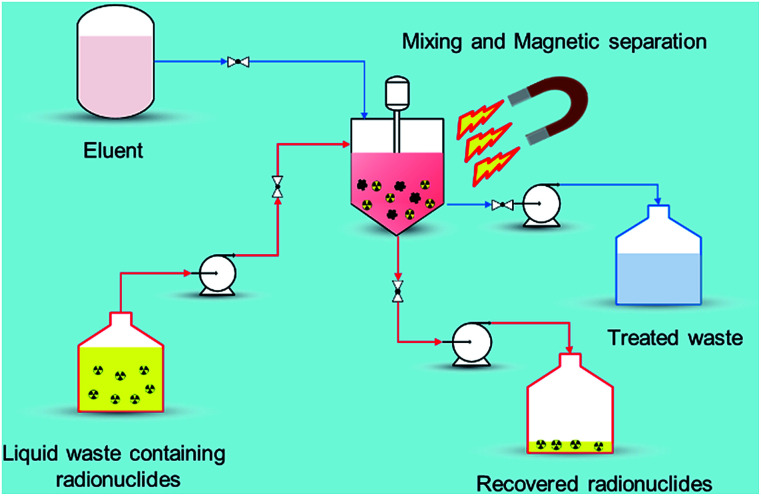
Magnetic assisted separation for radionuclides from wastewater.

Recently, nano-materials such as graphene oxide (GO), nano-scale zero valent iron (nZVI), self-assembled mono layers on mesoporous supports (SAMMS), carbon nanotubes (CNTs), zeolites, and nano-sized filtration membranes have been examined for the removal of radionuclides.^17^ All of these remedial alternatives have their pros and cons, for example, CNTs and GO have excellent ability to encapsulate various radionuclides with high adsorption capability but the methods to prepare them in bulk amount are difficult as well as expensive. Similarly, zeolites are the ecologically benign materials for removal of radionuclides but they suffer from poor stability due to Si and Al dissolution at alkaline pH range.^18^ Among the aforementioned established technologies, adsorption using magnetic nanoparticles (MNPs) under certain conditions has a definite edge (Fig. 2) over other methods due to its simplicity, effectiveness at removing dissolved contaminants in low concentration range (μg L^−1^ to mg L^−1^), high recovery, environment-benignity, and low maintenance cost.^19^ The main advantage of magnetic adsorbent and its facile separation is to reduce the radiation exposure to radiological worker in high radiation field so that the entire remediation process can be systematically regulated and controlled remotely. To treat the radioactive contaminants from aqueous waste streams, the MNPs need to be properly dispersed in a reactor or *in situ*. The extraction of radionuclides on MNPs can be achieved in a relatively short time right after collecting the MNPs using a magnet (or magnetic system). In comparison to conventional adsorbents, the radionuclides adsorbed on the MNPs can easily be tracked, retrieved, and reused with the aid of magnetic field which reduces the secondary pollution and protect the public especially during accidental release of radionuclides to the environment. *i.e.*, Fukushima, Chernobyl *etc.* Eventually, the final waste containing the concentrated radionuclides can be disposed of permanently in a safe storage area.

MNPs composed of magnetic metals include Fe, Ni, Co, and their oxides. Among these, magnetite (Fe_3_O_4_) nanoparticles (m-NPs), one of the most widely used magnetic materials, has gained immense attention due to its strong magnetic susceptibility, cost effectiveness, and biocompatibility. Owing to these exceptional features, m-NPs have a wide variety of application in diverse research disciplines such as magnetic resonance imaging, catalysis, separation, and environmental remediation. In the field of environmental remediation, several reviews have been documented on the removal of heavy metals,^20^ pesticides,^21^ and other emerging organic contaminants.^22^ Meanwhile, Maninder Kaur *et al.* studied the chelation processes of MNPs for the removal of minor actinides from spent nuclear fuel and demonstrated the design of effective processes.^23^ The reviews on the magnetite-based adsorbents for use in radioactive waste management is less well documented. This review aimed to bridge the knowledge gaps and to document essential researches in the field by presenting the recent decontamination studies of radionuclides by magnetite-based adsorbents, including their synthesis, and adsorption behavior of radionuclides between the adsorbent interfaces under diverse experimental conditions. In addition, case studies for the removal of radionuclides from real environmental samples were summarized to provide basic knowledge for optimizing their application in real-world remediation problems.

## 2. Synthesis of magnetite-based adsorbents

In the recent years, magnetite has been synthesized by many different methods such as co-precipitation,^24^ electrochemical deposition,^25^ synthesis in reverse micelles,^26^ combustion synthesis,^27^ mechano-chemical dispersion,^28^ sol–gel process,^29^ arc discharge,^30^ flow injection synthesis,^31^ hydrothermal,^32^ thermal decomposition,^33^ solvothermal,^34^ sonolysis,^35^ high-temperature annealing,^36^ and micro-emulsions.^37^ Features of these widely-adopted methods are presented in Table 1. Among the various synthesis routes, thermal decomposition as well as hydrothermal methods are found to be two of the most ideal ones based on particle size and well controlled morphology. In terms of biocompatibility and water solubility of m-NPs, co-precipitation method is frequently used but certain disadvantage such as uncontrolled shape, narrow size distribution, and aggregation of the particles could be associated for some applications. Some researchers have developed green/biological methods for the synthesis of m-NPs using seaweeds^38^ and microorganisms.^39^

**Table 1:** Pros and cons of different preparation methods for magnetite nanoparticles

Method	Pros	Cons	Ref.
Co-precipitation	■ Economical precursors	■ Broad size distribution	24
	■ Mild reaction conditions	■ Low reproducibility	
	■ Synthesis in H_2_O	■ Uncontrolled oxidation	
	■ Ease surface modification		
	■ Short synthesis time minutes per hours		
	■ Ease formation of ferrites		
	■ Ease conversion to g-Fe_2_O_3_		
	■ Ease scale-up		
Reverse micelle	■ Improved size control	■ Low reaction yield	26
	■ Narrow size distribution	■ Poor crystallinity	
	■ Ease size tunability	■ Surfactants are difficult to remove	
	■ Uniform magnetic properties		
Hydrothermal reaction	■ Improved size control	■ High pressure and reaction temperature	32
	■ Narrow size distribution	■ Safety of the reactants	
	■ Synthesis in H_2_O		
	■ Tunable magnetic properties		
Thermal/sonochemical decomposition	■ Narrow size distribution	■ Toxic organic solvents used	33 and 35
	■ High size control	■ High temperatures needed	
	■ High crystallinity	■ Phase transfer required	
	■ Possible scale-up	■ Mechanism is still under discussion	
	■ Tunable magnetic properties		
Sol–gel	■ Moderate temperature conditions	■ High pressure is required	29
	■ Relatively short reaction period	■ Usually needs expensive precursors	
	■ Good shape control	■ High permeability	
	■ Relatively narrow size distribution	■ Low wear resistance	
		■ Weak bonding	
Biological	■ High yield	■ Slow and laborious	38 and 39
	■ Low production cost		
	■ Good reproducibility		

## 3. Classification of magnetite-based adsorbents

**Fig. 3:**
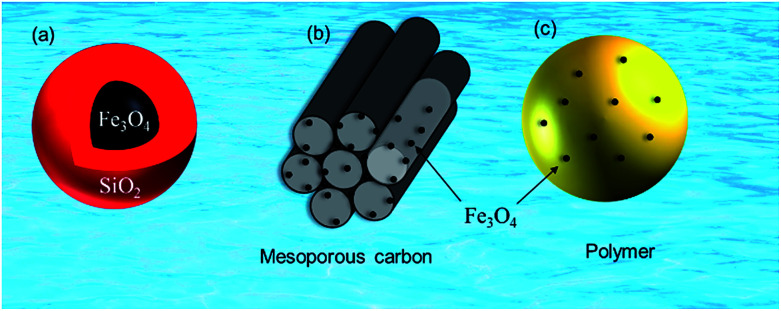
Magnetite-based adsorbents (a) silica protected magnetite, (b) mesoporous carbon supported magnetite and (c) magnetite embedded polymer.

**Fig. 4:**
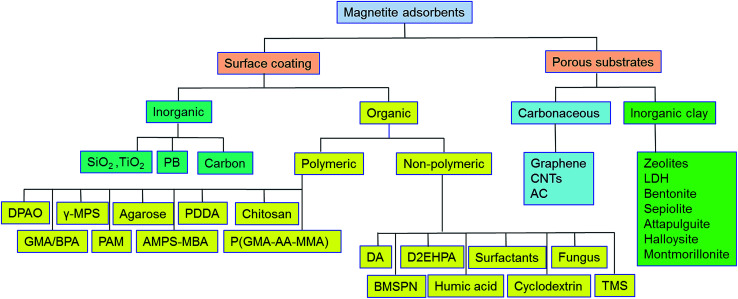
Classification of magnetite-based adsorbents for removal of radionuclides.

Pristine m-NPs containing the magnetic core are highly susceptible to aggregation that causes significant changes in magnetic properties. Moreover, m-NPs are prone to undergo autoxidation and potential Fe^2+^ leaching, and not well target-selective under ambient conditions.^40^ One elegant way of compensating for this problem involves modification of the magnetic core with the deposition of suitable chemical compounds which act as a protecting shell (Table 2). For its effective application to diverse contaminated sites, the chemical compounds for the m-NPs shell may be organic (*e.g.*, polymers and surfactants) or inorganic (*e.g.*, silica, carbon, and noble metals). As a result of proper modification, the resulting m-NPs exhibit higher stability and adsorption capacity by preventing potential oxidation and aggregation. In addition, the protecting chemical compounds can be further conjugated with various chelating agents, eventually making m-NPs a more versatile precursor for a broad range of environmental applications. Besides the surface modification or functionalization of m-NPs, an alternative approach can be achieved by *in situ* encapsulation of m-NPs with different substrate materials (porous carbonaceous materials, inorganic clay, *etc.*) to synthesize magnetically retrievable composites (Table 3). The resulting magnetic composites show remarkable performance for radionuclides removal which is simply unattainable by individual substrate components. Moreover, another strategy is to prepare magnetic polymer beads with homogeneously distributed m-NPs. Herein, m-NPs are dispersed in a in a polymeric solution and emulsified as a disperse phase. Then each droplet of the emulsion is transformed into bead either by cross-linking or evaporation.^41^ To date, magnetite-based composites have been fabricated with polymers, carbonaceous materials, biomaterials, and inorganic oxides for the enhanced removal of radionuclides (Fig. 3). Consequently, magnetite adsorbents can be categorized according to type of shell as well as type of porous substrate as given below (Fig. 4):

**Table 2:** Magnetite-based adsorbents for the removal of radionuclides

Adsorbent	Method	Protecting material	Functional group	Target	Temp	pH	*Q*_max_ (mg g^−1^)	Solution condition	Time	Mechanism	References
Fe_3_O_4_/quercetin	Co-precipitation	SiO_2_	Quercetin	U(vi)	298	5	12.3	DI	0.5 h		46
Fe_3_O_4_/SiO_2_	Co-precipitation	SiO_2_	—	U(vi)	298	6	52	0.01 M NaCl	3 h		92
MMSN10N	Co-precipitation	SiO_2_	Amino	U(vi)		5	160	DI	<2 h	Inner-sphere surface complexation	93
Fe_3_O_4_/SiO_2_	Co-precipitation	SiO_2_		Eu(iii)	298	7	37.9	DI			42
Fe_3_O_4_@SiO_2_/DTC	Co-precipitation	SiO_2_	DTC	Eu(iii)	298	7	11.8				42
Fe_3_O_4_@SiO_2_/APMS	Co-precipitation	SiO_2_	APMS	Eu(iii)	298	7	32.6				42
AO-Fe_3_O_4_@SiO_2_	Hydrothermal	SiO_2_	Amidoxime	U(vi)	298	5	105	0.01 M NaClO_4_	2 h	Inner-sphere surface complexation	43
Fe_3_O_4_@SiO_2_@APTES/PVA	Co-precipitation	SiO_2_	APTES/PVA	U(vi)	318	5	69	n.a	5 h		94
Sal–APS–FMNPs	Co-precipitation	SiO_2_	APS–salicylaldehyde	U(vi)		7	49	n.a			95
Fe_3_O_4_@SiO_2_@Ni-L	Hydrothermal	SiO_2_	Nickel–ethylene glycol	U(vi)	298	5	129.2	n.a	4 h	Inner-sphere complex by means of surface complexation with the Ni–O bond	96
Fe_3_O_4_	Co-precipitation	SiO_2_	Ammonium and phosphonate	U(vi)	—	9	70.7	DI	2 h	Electrostatic and chelating attraction	44
CB–MNs	Co-precipitation	SiO_2_	*p-tert*-butylcalix[4]arene	U(vi)				n.a			97
Fe_3_O_4_@SiO_2_@KTiFC	Solvothermal	SiO_2_	Potassium titanium ferrocyanide	Cs(i)	298		43	n.a	2 h	Ion exchange between H^+^ and Cs(i)	34
Fe_3_O_4_@SiO_2_@D*t*BuCH18C6	Hydrothermal	SiO_2_	D*t*BuCH18C6	Sr(ii)	298	1 M	9	1 M HNO_3_	10 h	Coordination with crown ether	98
PA–SMM	Hydrothermal	SiO_2_	DPTS	U(vi)	298	5	77	n.a	4 h	Strong chelation of the phosphonic group with uranium	99
MMSNs–PP	Co-precipitation	Mesoporous silica	Phosphonate	U(vi)	295	3.5	38	Artificial groundwater	144 h		47
Fe_3_O_4_@MS	Hydrothermal	Magnesium silicate		U(vi)	298	5.5	242.5	0.01 M NaClO_4_	5 h	At low pH; ion exchange, at high pH; inner surface complexation	48
Fe_3_O_4_@TiO_2_	Hydrothermal	TiO_2_		U(vi)	298	6	119	DI	4 h		100
Ketoxime–Fe_3_O_4_@C	Solvothermal	Carbon	Ketoxime	U(vi)	298	6	38.7	n.a	2 h		58
Hollow-Fe_3_O_4_@mC	Solvothermal	Mesoporous carbon		U(vi) Sr(ii) Eu(ii)	298	3	135, 117, 154	0.01 M NaClO_4_	2 h	Electrostatic	57
Fe_3_O_4_@K_2_ZnFe(CN)_6_	Co-precipitation	K_2_ZnFe(CN)_6_		Cs(i)	303		1965	n.a	2 h	Ion exchange	101
PB–Fe_3_O_4_	Co-precipitation	PB		Cs(i)	298	5.5	16.2	DI			102
Prussian blue-coated magnetic nanoparticles	Co-precipitation	PB		Cs(i)			96	DI	24 h		103
PB–MNC	Hydrothermal	PB		Cs(i)			45.8	n.a	6 h		54
MPB-1	Co-precipitation	PB		Cs(i)	298		146	DI	6 h		104
Magnetite PB	Co-precipitation	PB		Cs(i)	283	7	281	n.a	4 h		55
Fe_3_O_4_–O–CMK-3	Co-precipitation	Mesoporous carbon	Oxygen bearing groups	Cs(i)	298	6	205	DI	5 min	Electrostatic and ion exchange	24
Magnetic chitosan	Co-precipitation	Chitosan		U(vi)	300	5	42	DI	40 min		105
Amino acid functionalized chitosan magnetic nanobased particles	Co-precipitation	Chitosan	Alanine, serine	U(vi)	298	3.6	85.3, 116.5	n.a	50 min	At low pH, ion exchange; at mild acidic pH, chelation	63
EMMC	Co-precipitation	Chitosan	Ethylenediamine	U(vi)	303	3	83	DI	30 min		62
Amine-functionalized magnetic-chitosan nano-based particles	Co-precipitation	Chitosan	Diethylenetriamie	U(vi)	298	3.6	185.2	n.a	40 min	Anion exchange	106
Magnetic Schiff base chitosan	Co-precipitation	Chitosan	Schiff base	U(vi)	298	4	552	n.a	30 min		107
EDA–MCCS	Co-precipitation	Chitosan	Ethylenediamine	U(vi)	298	4.5	175.4	n.a	40 min	Chelation	108
TETA–MCS	Micro emulsion	Chitosan	Triethylene-tetramine	Th(iv)	298	4	133	n.a	60 min		109
IMCR	Co-precipitation	Chitosan		U(vi)	298	5	187.2	n.a	120 min	Chelation	65
MCS	Co-precipitation	Chitosan	Saw dust	Sr(ii)	293	9	12.6	n.a	30 min		110
Magnetic chitosan beads	Co-precipitation	Chitosan		Sr(ii)	303	8.2	11.6	n.a	6 h	–NH_2_ was mainly involved in Sr^2+^ adsorption	111
Magnetic chitosan composite particles	Precipitation	Chitosan		U(vi) Th(iv)	298	4, 5.5	667, 313	DI	2 h		64
PVA/chitosan magnetic beads	Co-precipitation	Chitosan	PVA	Co(ii)	303	6	14.39	DI		–NH_2_ and –OH was mainly involved in Co^2+^ adsorption	112
HCC–Fe_3_O_4_	Hydrothermal	Chitosan		U(vi)	298	7	263	n.a	3 h	Interaction of U(vi) with OH and NH_2_ groups	113
Fe_3_O_4_@agarose microsphere	Co-precipitation	Agarose		U(vi) Eu(iii)	298	5.2, 6	274, 194	0.01 M NaClO_4_	4 h		114
AAM cryobeads	Co-precipitation	Alginate Agarose		U(vi)	298	4.5–5.5	120.5	n.a			66
AO–Fe_3_O_4_/P(GMA–AA–MMA)	Co-precipitation	P(GMA–AA–MMA)	Amidoxime	U(vi)	298	4.5	201	DI	3 h	Complexation between U(vi) and amidoxime groups	67
PAMAMG_3_–Fe_3_O_4_/P(GMA–AA–MMA)	Co-precipitation	P(GMA–AA–MMA)	PAMAMG_3_	U(vi)	298	4.5	395	DI	1 h		115
Magnetic IIP	Co-precipitation	γ-MPS		U(vi)	298	4	1.1	n.a	45 min		69
DPAO–MNPs	Precipitation	DPAO		Th(iv)	298	4	666	n.a	150		116
Fe_3_O_4_@PAM	Hydrothermal	Polyacrylamide		U(vi)	293	5	221	0.01 M NaCl	24 h	Complexation between U(vi) and amide groups	70
Magnetic GMA/MBA	Co-precipitation	GMA/BPA	Ethylenediamine, diethylenetriamine	U(vi)	298	5	92, 158	DI		Coordination	71
Magnetite nanocomposite	Precipitation	AMPS–MBA	AO	U(vi)	298	4	476	n.a	150 min	Chelation between U(vi) ions and amidoxime functional groups	117
Magnetic Na–phlogopite	Co-precipitation	PDDA	Na–phlogopite	Cs(i)			69.7	DI		Ion exchange	118
Fe_3_O_4_@APTMS	Hydrothermal	SDS	APTMS	U(vi)	298	6	152	n.a		Complexation of U(vi) with surface amino groups	72
Magnetic oxine	Solvothermal	SDS	Oxine	U(vi)	298	7	125	n.a	>4 h	Inner-sphere surface complexation	73
Fe_3_O_4_–NH_2_		APTMS	NH_2_	U(vi)	298	5	269	DI	1 h		119
Fe_3_O_4_@cyclodextrin	Co-precipitation	Cyclodextrin		Eu(iii)	293	5	94.30	0.01 M NaNO_3_	3 h	At low pH, inner-sphere surface complexation. At high pH, precipitation and inner-sphere surface complexation	74
Fe_3_O_4_/BMSPN	Solvothermal	BMSPN		U(vi)	298	6	94.30	DI	6 h		120
Fungus-Fe_3_O_4_	Co-precipitation	Fungus		U(vi) Th(iv) Sr(ii)	303	5, 3, 5	281, 251, 101	Simulated wastewater 0.01 M NaClO_4_	48 h	Inner-sphere radionuclide complexes with oxygen-containing functional group	75
Fungus-Fe_3_O_4_	Co-precipitation	Fungus		U(vi)			171	n.a			121
Fe_3_O_4_–DA–BP				U(vi)		7		DI	30 min		122
Fe_3_O_4_	Co-precipitation	D2EHPA		U(vi)				Wastewater		Chelation	76
Fe_3_O_4_@HA	Co-precipitation	Humic acid		Eu(iii)	293	5	10.6	0.005 M NaCl	<30 min	Inner-sphere surface complexation	77

**Table 3:** Substrates of magnetite-based adsorbents for the removal of radionuclides

Adsorbent	Method	Substrates	Functional group	Target	Temp	pH	*Q*_max_ (mg g^−1^)	Solution condition	Time	Mechanism	References
CB[6]/GO/Fe_3_O_4_	Co-precipitation	GO	CB[6]	U(vi)	298	5	122.5	DI	2.5 h	Complexation with functional groups of CB[6]/GO/Fe_3_O_4_	82
Fe_3_O_4_/GO	Co-precipitation	GO		U(vi)	293	5.5	69.5	0.01 M KNO_3_	>4 h	At high pH: precipitation and inner sphere surface complexation	79
AOMGO	Co-precipitation	GO	Amidoxime	U(vi)	298	5	284.9	0.01 M NaClO_4_	2 h	Inner sphere surface complexation	123
MnO_2_–Fe_3_O_4_–rGO	Hydrothermal	rGO	MnO_2_	U(vi)	328	6	108.7	n.a	6 h	Surface complexation, cation exchange and electrostatic interaction	124
AMGO	Hydrothermal	GO	Amino	U(vi)	298	5.9	141.2	DI	100 min	Interaction with nitrogen- and oxygen containing functional groups	125
Fe_3_O_4_/GO	Hydrothermal	GO		U(vi)	298	5.9	283.2	0.01 M NaNO_3_	5 h		126
Fe_3_O_4_/GO	Co-precipitation	GO		Cs(i) Sr(ii)	293	5	15.8 38.4	0.01 M NaCl	24 h	H^+^/Na^+^ exchange	127
MGO	Co-precipitation	GO		Eu(iii)				NaClO_4_ and N_2_	5 h	Inner-sphere surface complexation	128
Magnetic GOs	Co-precipitation	GO		Eu(iii)	293	4.5	70.2	0.01 M NaClO_4_	24 h	Inner-sphere surface complexation	129
M/GO	Co-precipitation	GO		Sr(ii)	303	8.5	9.8	0.01 M NaClO_4_	2 h	Inner-sphere surface complexation	130
Magnetic graphene oxides	Co-precipitation	GO		Sr(ii) Cs(i)	293	4	14.7 9.3	0.01 M NaClO_4_	24 h	Cation exchange and inner-sphere surface complexation	131
PB/Fe_3_O_4_/GO	Co-precipitation	GO	PB	Cs(i)	298	7	55.6	DI	24 h	H^+^ exchange and/or ion trapping	80
PFGM	Co-precipitation	GO/calcium alginate	PB	Cs(i)	298	7	43.5	DI	24 h	K^+^/H^+^ exchange and or ion trapping	132
Fe_3_O_4_@C@Ni–Al LDH	Co-precipitation	Carbon	Ni–Al LDH	U(vi)	298	6	174	n.a	3 h	Surface adsorption and intercalation	133
MMWCNTs	Co-precipitation	CNTs		Th(iv)	298	4.1	0.232	n.a	40 h	Surface complexation	134
Fe_3_O_4_/AC	Co-precipitation	AC		Sr(ii)	303	5	42.3	0.01 M NaCl	4 h		135
PAF magnetic adsorbent	Co-precipitation	AC	Polyethylenimine	U(vi)	293	5	115.3	n.a	1 h		136
CD/HNT/iron oxide	Co-precipitation	HNT	CD	U(vi)	298	5.5	107.6	0.01 M NaNO_3_	4 h	At high pH: inner-sphere surface complexation, at low pH electrostatic or outer sphere complexation	85
HNTs–Fe_3_O_4_	Co-precipitation	Halloysite nanotubes		U(vi)		5.5	88.3	0.01 M NaCl	12 h	Ion exchange and surface complexation	137
MZC	Co-precipitation	Zeolite		Sr(ii) Cs(i)	298	8	83.7207.4	n.a	>2 h	Ion exchange	138
MZNC	Co-precipitation	Zeolite		Sr(ii) Cs(i)	298	8	89, 229	n.a	>30 min	Ion exchange	139
Magnetic 4A zeolite	Hydrothermal	Zeolite		Cs(i)	298		106.6	n.a	48 h		140
Attapulgite-iron oxide	Co-precipitation	Attapulgite		Eu(iii)	293	5	117	0.01 M NaClO_4_	24 h	At low pH: outer sphere surface complex, ion exchange, at high pH: inner sphere surface complex, surface precipitation	86
Magnetic citrate Mg–Al LDH	Co-precipitation	Mg–Al LDH	Citrate	U(vi)	298	6	180	n.a	4 h	Formation of chelate complex	88
CMLH	Co-precipitation	LDH/hydroxyapatite		U(vi)	298	6	208	n.a	1 h	Surface adsorption or complexation	141
CS-g-MB	Solvothermal	Bentonite	Chitosan	Cs(i)		7.61	149	Seawater	24 h	Ion exchange with chitosan functional groups such as OH groups	89
Silicate-based multifunctional nanostructured materials with magnetite and prussian blue	Co-precipitation	Sepiolite	PB	Cs(i)	295		102	n.a	3 h		87
MMT/Fe_3_O_4_	Co-precipitation	Ammonium-pillared montmorillonite		Cs(i)	298	6.7	27.5	n.a	1 h	NH_4_^+^ ion exchange and surface hydroxyl group coordination	142

### 3.1 Surface coated magnetite-based adsorbents

#### 3.1.1 Inorganic coating

##### 3.1.1.1 Silica coating

Surface protection of the magnetite particles by a silica layer is the most commonly-employed method due to several advantages including chemical and colloidal stabilities, low cost, and controlled porosity.^23^ In addition, the terminal OH groups on the silica surface can be easily functionalized with various organic and inorganic moieties with respect to selective removal of metals. Silica coating can be achieved through Stober process *via* sol–gel reaction which involves the use of tetraethyl orthosilicate (TEOS) in acidic and/or basic pH conditions under vacuum. Several works have been reported in the literatures demonstrating the effective use of silica protected m-NPs grafted with various functional groups for the removal of radionuclides,^42,44^. However, the –Si–O–Si– bond in silica coating is susceptible to rapid degradation in basic conditions resulting in significant loss of surface-grafted functional groups.

A comparison study of Eu(iii) adsorption was conducted with four different types of magnetic adsorbent materials including bare magnetite (Fe_3_O_4_), silica-coated magnetite (Fe_3_O_4_@SiO_2_), and silica-coated magnetite with surfaces functionalized with amine groups (Fe_3_O_4_@SiO_2_/APMS) and dithiocarbamate groups (Fe_3_O_4_@SiO_2_/DTC).^42^ As a result of subsequent modification, the net surface charge was significantly varied from 2.2 to −44 mV. These materials adsorbed Eu in the order of Fe_3_O_4_@SiO_2_ ≈ Fe_3_O_4_@SiO_2_/APMS > Fe_3_O_4_ > Fe_3_O_4_@SiO_2_/DTC at pH 7 in DI water; higher adsorption was due to the preferred coordination of Eu(iii) with the O donors. The Eu adsorption was lower in Fe_3_O_4_@SiO_2_/DTC because of insufficient accessible O donating ligands than bare Fe_3_O_4_.

Magnetic adsorbents comprising of magnetic core protected with silica shell and amidoxime as a functional group were synthesized and investigated to extract uranium from aqueous solutions.^43^ In comparison with raw silica coated magnetite (Fe_3_O_4_@SiO_2_), the prepared Fe_3_O_4_@SiO_2_–AO exhibited better adsorption capability due to the strong complexation of U(vi) with amidoxime moieties. The maximum adsorption capacity obtained by this adsorbent was 105 mg g^−1^ at pH 5 in 0.01 M NaClO_4_ solution. For the pH values below 3, retention capacity significantly dropped, which meant that the adsorbed U(vi) could be desorbed under acidic conditions. For this reason, the stability of adsorbent was assured by dispersing the adsorbent in hydrochloric acid solution in the concentration range of 0.01–2 mol L^−1^ up to 2 weeks and then a regeneration study was performed where the adsorbent was used for six consecutive runs with only a 6.2% decline in adsorption efficiency. In addition to good stability and better adsorption capacity, the adsorption process was rapid and reached the equilibrium within 2 h.

Post-synthesis grafting method was adopted to prepare a magnetic adsorbent bi-functionalized with ammonium and phosphonate groups for the removal of U(vi) in an alkaline environment ^44^5. The combination of dual functionalities enhanced adsorption performance and maximum U(vi) uptake capacity was 70.7 mg g^−1^ at pH 9 in DI water due to electrostatic attraction between NH_4_^+^ and (UO_2_)_3_(OH)_7_^−^, and complexation between phosphonate and U(vi). Unlike NO_3_^−^ and SO_4_^2−^ anions, the presence of PO_4_^3−^ as a co-existing anion inhibited the adsorption process due to possible complexation between U(vi) and dissociated PO_4_^3−^. Considering the minute U(vi) adsorption at pH 4, the adsorbent was renewed with 0.2 mol L^−1^ HNO_3_ for six cycles where the U(vi) recovery decreased from 97.1% to 90.4%.

Quercetin (3,3′,4′,5,7-pentahydroxyflavone) is a naturally occurring dietary flavonoid that has the ability to form stable complexes with transition metals (Fe, Co, Ni, Cu, and Zn).^45^ In this context silica-coated Fe_3_O_4_ nanoparticles were modified with quercetin and employed as a adsorbent for the uptake of uranyl ions (UO_2_^2+^) in spiked ground and commercial (Nestle) drinking mineral water samples.^46^ This adsorbent exhibited the maximum uptake capacity of 12.3 mg g^−1^ at pH 5 in DI water. In addition, the adsorption process was rapid and reached steady state within thirty minutes.

Li *et al.*^47^ anchored various organic moieties including aminopropyl, benzoylthiourea (BT), dihydroimidazole (DIM), polyaryloamidoxime (AD), phosphonate (PP), phosphonate-amino (PPA), chloropropyl, poly(propylenimine) (PPI), and poly(amidoamine) (PAMAM) into mesopores of thickness 3 nm of magnetic mesoporous silica particles (MMSNs) and explored the reactivity of each functionalized adsorbent towards uranium in low and high pH artificial groundwater. Under the acidic conditions (pH 3.5), MMSNSs–PP was found to be the best adsorbent with highest U(vi) adsorption capacity (37.5 mg g^−1^). However, in alkaline conditions (pH 9.6), MMSNs–PPI exhibited the highest U(vi) adsorption capacity (133.3 mg g^−1^).

Yolk–shell microspheres with Fe_3_O_4_ cores and hierarchical magnesium silicate shells (Fe_3_O_4_@MS) have been successfully synthesized by combining the versatile sol–gel process and hydrothermal reaction in which, Fe_3_O_4_@SiO_2_ served as a chemical template.^48^ The as prepared Fe_3_O_4_@MS microspheres were assessed as a potential adsorbent for U(vi) removal from water. The U(vi) adsorption on Fe_3_O_4_@MS microspheres was strongly dependent on pH and the ionic strength. The maximum adsorption capacity for U(vi) was calculated to be 242.5 mg g^−1^ at pH 5.5 in 0.01 M NaClO_4_ solution. The thermodynamic analysis revealed that the adsorption of U(vi) onto Fe_3_O_4_@MS was spontaneous and endothermic. In addition, the adsorption of U(vi) was dominated by ion exchange at low pH conditions, but by the inner-sphere surface complex at high pH (>6).

Among the various radionuclides, Tc is comparatively a less studied radionuclide due to difficulty in removing high mobile Tc in the environment. To prevent migration of Tc, most Tc-bearing radioactive waste should be solidified using waste form like glass. However, at glass vitrification temperatures (∼1200 °C), Tc is rapidly volatilized resulting in the substantial loss of Tc from the final waste glass. Recently scientists found that addition of magnetite as an additive during the vitrification process significantly improved the Tc retention in glass waste form, where it is incorporated in the octahedral sub-lattice provided by magnetite.^49,50^ In addition, quantum-mechanical modeling techniques were used to demonstrate incorporation energies and optimized lattice bonding environments for charge-balanced Tc(iv) incorporation mechanisms in magnetite.^49^

##### 3.1.1.2 Prussian blue coating

Prussian blue (PB) is a low-cost cyano-bridged coordination polymer containing hexacyanometallates and transition metal ions with chemical formula Fe_7_(CN)_18_. It has been used as an antidote for patients contaminated with radioactive cesium since the Chernobyl nuclear accident in 1986. The high binding selectivity arises from size compatibility of hydrated cesium ion with the cage size of the PB lattice.^51^ This is why a majority of studies are based on the Prussian blue nanoparticles (PB-NPs) for cesium decontamination from the actual environmental samples including seawater and soil. However, the Cs ions are incorporated in PB lattice structure therefore, it is not easy for the PB-NPs to be regenerated. In addition, the use of PB-NPs and its analogues might be problematic at high pH due to leaching of cyanide ions. More importantly, the retrievability of Cs loaded PB-NPs is a hurdle in the real field applications. Sasaki and Tanaka^52^ reported the synthesis of Prussian-blue-modified magnetite (PB-Fe_3_O_4_) through a scalable co-precipitation method for the decontamination of aqueous Cs. Consequently, the maximum adsorption amount of Cs with PB-Fe_3_O_4_ was 16.2 mg g^−1^ and the adsorption capacity was not greatly influenced in the presence of high concentration of NaCl solution. Thammawong *et al.*^53^ developed magnetic nano-adsorbent using a facile chemical co-precipitation of ferric and ferrous salts to firstly obtain the MNP cores, which are further reacted with [Fe(CN)_6_]^4−^ under acidic conditions to produce a coating of the PB layer onto the MNPs. The adsorbent possessed both high Cs adsorption capacity (96 mg Cs g^−1^ adsorbent) and large distribution coefficient, *K*_d_ (3.2 × 10^4^ mL g^−1^ at 0.5 ppm Cs in DI water). The magnetic adsorbent was proposed to use as a new type of Cs decorporation drug for Cs contaminated patients.

Later, Yang *et al.*^54^ fabricated the Prussian blue-functionalized magnetic nanoclusters (PB-MNC) by hydrothermal method without addition of coating materials such as PDDA, to provide much higher saturation magnetization (27.5 emu g^−1^) and to coat large quantities of PB on the surface of the MNC. As a result, PB-MNC had a large Cs distribution coefficient, even in the presence of 3000 ppm competing ions such as K^+^, Na^+^, Ca^2+^, and Mg^2+^, and excellent removal efficiency (>99.7%) of radioactive cesium from contaminated water.

Recently, magnetite PB adsorbent was synthesized by binding PB to a core of magnetite (Fe_3_O_4_) nanoparticles for highly efficient and rapid separation of Cs from aqueous solution.^55^ The adsorbent showed a maximum Cs adsorption capacity of 280.82 mg g^−1^ at an initial Cs concentration of 50 mM at pH 7, and 10 °C, which is much higher than those of previously reported PB-based adsorbents for removing Cs from the various solution. The higher adsorption capacity was positively correlated with large surface area of 322.2 m^2^ g^−1^ of the magnetite–PB nanocomposite.

##### 3.1.1.3 Carbon coating

Carbon-coated magnetite nanoparticles are of great significance due to their better stability for an oxidative degradation as compared to silica coating. There are various approaches including chemical vapor deposition (CVD), pyrolysis, and detonation-induced reaction for the carbon coating.^56^ The main advantage of CVD is the precise control on the thickness of carbon coating but this require high cost due to the need to use complex equipment and high energy consumption. On the other hand, the pyrolysis approach is an attractive option due to bulk production of carbon coated nanomaterials.

Hollow Fe_3_O_4_ magnetite nanoparticles coated with mesoporous carbon (h-Fe_3_O_4_@mC) have been synthesized using silica nanospheres as the sacrificial matrix and investigated as possible adsorbent for removal of radionuclides.^57^ Effects of contact time, pH, and initial concentrations on the interaction of h-Fe_3_O_4_@mC with U(vi), Eu(iii), Co(ii), and Sr(ii) have been studied. The dependence of adsorption on pH is relevant to both the surface properties of adsorbents and the relative distribution of radionuclides species in solutions. As a result of mesoporous structure, carboxyl-functionalized surface, and low density due to hollow cavity, h-Fe_3_O_4_@mC shows efficient adsorption for radionuclides even in acidic solutions. The maximum adsorption capacities of U(vi), Eu(iii), Co(ii), and Sr(ii) on h-Fe_3_O_4_@mC at pH 3.0 in 0.01 M NaClO_4_ solution calculated from the Langmuir isotherm model were 0.566, 1.013, 0.860, and 0.733 mmol g^−1^, respectively.

The synthesis of Ketoxime-functionalized carbon coated iron oxide (Fe_3_O_4_@C–KO) was conducted by Liu *et al.*^58^ for the removal of hexavalent uranium from water. The resulting magnetic adsorbent showed U(vi) adsorption capacity of 38.7 mg g^−1^ at pH 6 in DI water. The adsorption–desorption experiment was repeated for three cycles with 0.5 M HCl resulting in >80% desorption efficiency.

Recently, Husnain *et al.*^24^ developed three magnetic mesoporous carbon adsorbents, *i.e.*, Fe_3_O_4_–O–CMK-3, O–Fe–CMK-3 and Fe–O–CMK-3 using co-precipitation, impregnation, and co-casting methods, respectively. These materials adsorbed Cs in the order of Fe_3_O_4_–O–CMK-3 > O–Fe–CMK-3 > Fe–O–CMK-3; higher adsorption was due to the presence of polar groups on the surface of the adsorbent. The Fe_3_O_4_–O–CMK-3 removed Cs effectively without leaching of Fe, and could be collected within a few seconds by using a magnet. Transmission electron microscopy (TEM) analysis confirmed the formation of a 20 nm thick oxidized mesoporous carbon coating around the m-NPs. The adsorbent showed maximum Cs adsorption capacity of 205 mg g^−1^ at pH 6 in DI water with good adsorption affinity even in the presence of high concentrations of interfering cations (K^+^, Na^+^, Li^+^, Ca^2+^, and Sr^2+^). The Cs adsorption onto Fe_3_O_4_–O–CMK-3 was due to a synergistic effect of electrostatic interaction and ion exchange of H^+^ for Cs. The Fe_3_O_4_–O–CMK-3 adsorbent was regenerated well, and could be used for six adsorption cycles, unlike existing PB containing magnetic adsorbents.

#### 3.1.2 Organic coating

##### 3.1.2.1 Polymeric coating

The presence of different functional groups (COOH, SO_4_^2−^, PO_4_^3−^) in polymers can be chemically linked or physically adsorbed on the surface of magnetite particles such that the polymers surround the magnetite particles with a protective layer.^59^ The thickness of the layer can be tuned by the solution pH.^60^ Repulsive forces are generated due to this layer which balances the magnetic and the van der Waals attractive forces acting on the NPs. Because of this layer, particles are well dispersed in solution. However, in acidic solutions, this layer is rapidly deteriorated resulting in the leaching of the magnetic core. In addition, polymer layers are not stable at high temperatures.

Chitosan is a naturally abundant, low cost ecologically benign biopolymer.^61^ It can bind various metals due to the presence of a high percentage of amino (NH_2_) groups which makes it a versatile adsorbent. Recently, ethylenediamine-modified magnetic chitosan (EMMC) complex was developed by Wang *et al.*^62^ as a novel magnetic adsorbent for U(vi) removal. Infra-red (IR) analysis demonstrated that Fe_3_O_4_ particles were successfully bound by chitosan and more amino groups appeared in the EMMC samples. EMMC was found to be quite efficient at adsorbing uranyl ions in the pH range of 2 to 7 in DI water. Adsorption equilibrium was established within 30 min, and the kinetic experimental data was in good agreement with those estimated by pseudo-second-order kinetic model, suggesting that the chemical adsorption was the rate-limiting step. The adsorption data could be best interpreted by the Sips model with a maximum adsorption capacity of ∼83 mg U g^−1^. The EMMC could be regenerated by 0.1 M NaOH solution.

Another suitable modification concerns the synthesis of hybrid materials composed of a magnetic core with a chitosan coating that was functionalized by amino acids (alanine/serine) through crosslinking epichlorohydrin for the uranium recovery from dilute solutions.^63^ Both functionalized adsorbents efficiently captured uranyl at pH 3.6. The adsorption mechanism mainly included ion exchange of anionic uranyl-sulfate species including UO_2_(SO_4_)_2_^2−^ and UO_2_(SO_4_)_3_^4−^ at pH < 3.6, whereas chelation with amino groups and carboxylate groups at pH = 6.7. Based on the Langmuir model, the maximum U(vi) adsorption capacities were 85.3 mg g^−1^ and 116.5 mg g^−1^ for magnetic nano particles with alanine and serine functionalized chitosan, respectively. Due to poor stability of the chitosan biopolymer in acidic conditions, desorbing solution contained 0.5 M urea dissolved in slightly acidic aqueous solutions (few drops of 0.2 M H_2_SO_4_, 2 < pH < 3). Consequently, after five successive cycles, adsorption efficiency was close to 93% for alanine-based adsorbent and 91% for serine-based adsorbent.

Magnetic iron chitosan composite particles with 40 μm average size were obtained by *in situ* procedure and investigated as an economical scavenger for the radioactive wastewater contaminated with U(vi) and Th(iv).^64^ The results indicated that the magnetic composite had superior adsorption capacities for both uranyl ions (666.67 mg g^−1^ at pH 4) as well as for thorium ions (312.50 mg g^−1^ at pH 5.5) compared to other low-cost adsorbents reported in the literature. Although, saturated magnetization of the composite declined from 24 to 18 emu g^−1^ during the adsorption process. However, this value (18 emu g^−1^) was sufficient for magnetic separation and recovery of radionuclide loaded adsorbent. According to thermodynamic studies, the adsorption process was spontaneous and endothermic. In addition, both the radioactive cations could be recovered by acidic desorbing agents (hydrochloric acid and nitric acid).

A chemically cross-linked U(vi)-imprinted magnetic chitosan resin (IMCR) was synthesized by using the ion-imprinting method with U(vi) as a template and glutaraldehyde as a cross-linker, respectively.^65^ Moreover, the non-imprinting magnetic chitosan resin (NIMCR) was synthesized as a control. The monolayer adsorption capacity was 187.26 mg g^−1^ for IMCR and 160.77 mg g^−1^ for NIMCR at pH 5.0 in DI water, respectively. The IMCR showed better adsorption capacity for the U(vi) removal than NIMCR due to more active adsorption sites resulting from a large number of size compatible cavities for uranyl ions by ion imprinting. Thermodynamic studies suggested that the adsorption process was spontaneous and exothermic. Finally, the U(vi) loaded resin could be regenerated for repeated use with 0.5 M HNO_3_.

The novel development of alginate–agarose–magnetite cryobeads by the process of cryotropic–gelation at subzero-temperature was carried out for the recovery of hexavalent uranium from the aqueous sub-surfaces.^66^ Due to high interconnected porosity (∼90%), these cryo-beads exhibited lower density resulting in their excellent floating ability in aqueous medium. Rheological analysis of cryo-beads revealed its stability and increased stiffness after uranium adsorption. The maximum uranium adsorption (97%) was observed at an initial U(vi) concentration of 100 mg L^−1^ at the pH range of 4.5–5.5 in DI water. The 0.1 M HCl solution was found to be an efficient eluent for the uranium desorption. This biosorbent showed ∼70% of uranium recovery after five repeated cycles for the uranium desorption. In addition, only 50% of U(vi) adsorption was observed in natural seawater after contact time of one day due to the weak cryogel–uranyl ion complexation in the presence of seawater components.

The synthesis of magnetic adsorbent (AO–Fe_3_O_4_/P(GMA–AA–MMA)) was performed by grafting amidoxime groups onto the surface of superparamagnetic polymer microspheres synthesized by a novel controlled radical polymerization technology using 1,1-diphenylethylene (DPE) as radical controlling agent.^67^ The prepared magnetic adsorbent was applied to adsorb uranium(vi) from aqueous solutions. An optimum U(vi) adsorption capacity of 200.5 mg g^−1^ was obtained at pH 4.5 in DI water. The adsorption of U(vi) on the magnetic adsorbent was mainly attributed to surface complexation *via* the coordination of U(vi) ions with amidoxime groups. The adsorbent could also selectively adsorb U(vi) in aqueous solution containing co-existing ions efficiently. Moreover, the desorption studies showed (AO–Fe_3_O_4_/P(GMA–AA–MMA)) could be used repeatedly and adsorption capacity did not have any noticeable loss after five cycles.

In the recent times, magnetic adsorption based on molecular recognition in molecularly imprinted polymers (MIPs) has drawn considerable attention owing to high selectivity for target ion, excellent reusability as well as high stability.^68^ Based on this ion imprinted polymer (IIP) embedded with γ-methacryloxypropyltrimethoxysilane (γ-MPS) coated magnetic particles were synthesized by bulk polymerization for selective extraction of uranyl.^69^ The U(vi) adsorption capacity of the magnetic polymers was found to be 1.1 and 0.95 mg g^−1^ for the IIP and its control ion non-imprinted polymer (NIP), respectively. The optimum time to reach equilibrium was 45 min. Studies from binary mixtures of metal ions in aqueous solutions exhibited that the magnetic adsorbent selectivity followed the order: U(vi) > Ni(ii) > Pb(ii). In addition, the synthesized products were tested in the mine wastewater sampled from Germiston (South Africa) with a pH 2.6, ORP 436 mV, and conductivity 680 μS cm^−1^. The mining site was previously engaged in open cast and deep mining activities in close vicinity of a water source. The sample contained Au (0.27 ppm), Co (22.95 ppm), Cr (0.83 ppm), Cu (11.25 ppm), Fe (1.8 ppm), Hg (0.67 ppm), Mn (72 ppm), Ni (48.3 ppm), U (8.5 ppm), and Zn (54.75 ppm). Approximate U(vi) recoveries of 77% and 66% using the magnetic IIP and NIP, respectively, were recorded. The removal efficiency of magnetic IIP was higher than magnetic NIP due to the imprinting effect. Stability and reusability of the magnetic polymers were monitored up to the sixth cycle without significant loss in extraction ability.

Water soluble, polyacrylamide coated magnetite Fe_3_O_4_@PAM composites were prepared by *in situ* polymerization technique and tested as adsorbent to adsorb U(vi) ions from water by using batch adsorption technique.^70^ The adsorption isotherms were well fitted by the Langmuir adsorption model, and the maximum U(vi) adsorption capacity of Fe_3_O_4_@PAM at pH 5.0 in 0.01 M NaCl solution was around 221 mg g^−1^. Based on the X-ray photoelectron spectroscopy (XPS) analysis, the nitrogen-containing functional groups on the surface of Fe_3_O_4_@PAM were involved in complex formation with U(vi). Acidic solution (0.02 M HCl) could be used to desorb the loaded U(vi) in 24 h, and the adsorption ability of regenerated composite remained >90% even after 5 cycles.

Magnetic glycidyl methacrylate resin particles with nano-magnetite core and glycidyl methacrylate/*N*,*N*′-methylene-bis-acrylamide (GMA/MBA) resin shell were prepared and immobilized with ethylenediamine and diethylenetriamine for removal of U(vi) in water samples.^71^ The maximum U(vi) adsorption capacities on R-1 and R-2 were recorded to be 92 and 158 mg g^−1^, respectively, at pH 5 in DI water. The kinetic results revealed that the pseudo-second-order adsorption was the predominant mechanism. Hexavalent uranium removal efficiency for both resins increased as temperature increased showing the endothermic nature of the adsorption process. Among the various applied isotherm models including Langmuir, Freundlich, Temkin, and Dubinin–Radushkevich, the adsorption reaction was best correlated with Langmuir model. To have real application of these synthesized resins, uranium was extracted successfully from three granite samples collected (from Gabal Gattar pluton, North Eastern Desert, Egypt) after the wet acidic digestion of granite samples. The studied resins showed good durability and regeneration using HNO_3_.

##### 3.1.2.2 Non-polymeric coating

Surfactants form self-aggregates by the immobilization or by the physical adsorption at the surface of magnetic core and protect it from oxidation by creating a shell structure around it. In this context, the anionic surfactant sodium dodecyl sulfate (SDS) coated Fe_3_O_4_ particles were synthesized and further functionalized with organosiloxanes (labeled as Fe_3_O_4_@APTMS).^72^ The as prepared adsorbent showed high adsorption affinity of 151.80 mg^−1^ at pH 6 towards U(vi) which was expected due to complexation reaction between surface amino groups and U(vi). The results revealed that the presence of interfering ions such as Na^+^, K^+^, Ca^2+^, Sr^2+^, and Mg^2+^ had no significant effect on adsorption of U(vi) on Fe_3_O_4_@APTMS. The adsorbent was readily regenerated by 0.1 M NaOH and reused for three times.

Surfactant-coated ferroferric oxide immobilized with oxine functionality was prepared and used as magnetic adsorbent for U(vi).^73^ The U(vi) adsorption was strongly dependent on pH and independent of ionic strength, signifying that the adsorption was due to an inner-sphere surface complexation. The 0.2 mol L^−1^ HCl solution was used as a desorbing solution for the regeneration experiments, and the reusability of the magnetic oxine was decreased from 82% to 78% in three adsorption–desorption cycles.

The Fe_3_O_4_@CD MCs were synthesized by using a simple chemical co-precipitation method for the removal of Eu(iii) from aqueous phase.^74^ In comparison to Fe_3_O_4_, the prepared Fe_3_O_4_@CD MCs demonstrated a higher adsorption capacity toward Eu(iii). The adsorption kinetics of Eu(iii) on Fe_3_O_4_@CD MCs could attain equilibrium within 3 h. The pH-dependent and ionic strength-independent Eu(iii) adsorption on the surface of Fe_3_O_4_@CD MCs suggested that the adsorption mechanism of Eu(iii) was inner-sphere surface complexation at low pH, whereas the removal of Eu(iii) was achieved by simultaneous precipitation and inner-sphere surface complexation at pH >6.8. The Langmuir and Freundlich models were employed to simulate adsorption isotherms for Eu(iii) on Fe_3_O_4_@CD MCs. Although the adsorbent had low cost with high removal efficiency, it did not regenerate, which may limit its real application potential.

Furthermore, the bio-nanocomposite with uniform decoration of m-NPs on a fungus surface was synthesized by a self-assembly technique. This bio-nanocomposite was then used to adsorb radionuclides such as Sr(ii), Th(iv) and U(vi) in water.^75^ These m-NPs were bound to fungus surface by means of chemical bonds as evidenced by Fourier transform infrared spectroscopy (FTIR). According to the Langmuir isotherm model maximum adsorption capacities of fungus-Fe_3_O_4_ were 100.9, 223.9 and 280.8 mg g^−1^ for Sr(ii) and U(vi) at pH 5.0, and Th(iv) at pH 3.0, in 0.01 M NaClO_4_ solution respectively, around 303 K. XPS analysis predicted the formation of inner-sphere radionuclide complexes due to bonding with oxygen-bearing functionalities (*i.e.*, alcohol, acetal and carboxyl) of fungus-Fe_3_O_4_. The thermodynamic parameters confirmed that the adsorption of radionuclides on fungus-Fe_3_O_4_ was a spontaneous and endothermic process. Moreover, it was noticed that fungus-Fe_3_O_4_ could be regenerated with 0.1 M HCl desorbing solution and reused at least five times.

Another interesting study involves the use of organophosphorus solvent, Di-(2-ethylhexyl) phosphoric acid (D2EHPA), coated onto magnetic nanoparticles by simple mixing and drying and the resulting surface-coated adsorbent was used for removal of U(vi) from the raffinate solution of Isfahan's Uranium conversion plant.^76^ The solution contained diverse cations (U (23 ppm), Na (4360 ppm), Al (0.32 ppm), Ni (2 ppm), Bi (0.2 ppm), Nb (0.1 ppm), Ca (0.1 ppm)) and anions (F^−^ (18 755 ppm), NO_3_^−^ (4870 ppm), SO_4_^2−^ (1648 ppm), Cl^−^ (210 ppm)). After the adsorption of uranium, the final concentration of U(vi) was lowered to 0.7 ppm in the waste such that it could be safely disposed in accordance to EPA and Nuclear Regularity Commission (NRC) rules. The optimum uranium recovery was obtained when 25% w/w D2EHPA/m-NPs were used in 0.5 M HNO_3_ solution.

Humic acids, the major organic constituents of soil are produced by biodegradation of dead organic matter. It is a complex mixture of many different acids containing carboxyl and phenolate groups. Humic acids can form stable complexes with ions that are frequently found in the natural environment creating humic colloids. In view of this point, environmentalists synthesized humic acid-coated Fe_3_O_4_ magnetic nanoparticles (Fe_3_O_4_@HA MNPs) as an adsorbent to evaluate its removal efficiency towards Eu(iii) under various environmental conditions.^77^ The kinetic studies showed that Eu(iii) adsorption onto Fe_3_O_4_@HA MNPs could attain equilibrium within 30 min and 99% Eu(iii) adsorption was observed at pH 8.5 with an initial Eu(iii) concentration of 3 mg L^−1^ in different solutions including 0.05 M NaNO_3_, 0.05 M NaCl, 0.05 M Na_2_SO_4_, and 0.05 M Na_2_HPO_4_. The rapid kinetics and high removal efficiency was positively correlated with the abundant surface sites provided by the coated HA macromolecules. The removal of Eu(iii) was dominated by inner-sphere surface complexation. The synthesized adsorbent showed good recycling performance up to six cycles using 0.01 M HCl solution as an eluent. Based on the negligible leachability of Eu from the loaded adsorbent in tap water over a period of three months, the Fe_3_O_4_@HA MNPs was proposed as a highly effective material for the enrichment and pre-concentration of radionuclide Eu(iii) or other trivalent lanthanides/actinides in geological repositories or in nuclear waste management.

### 3.2 Substrates of magnetite-based adsorbents

#### 3.2.1 Carbonaceous substrates

Owing to high specific surface areas, chemical inertness, biocompatibility and thermal stability, carbonaceous substrates have gained enormous attention in environmental remediation. In addition, carbonaceous substrate provides stable sites for magnetite nanoparticles loading to prevent their oxidation and aggregation. Various kinds of magnetic composites containing porous carbon such as activated carbon, multi-walled carbon nano tubes, mesoporous carbons, and graphene oxide have been synthesized to remove radioactive contaminants from aqueous solutions. Among these composites, graphene oxide (GO) based magnetite composites have received the most research interest owing to their outstanding physicochemical properties of GO.^78^

In 2013, the chemical co-precipitation strategy was adopted to synthesize magnetic graphene/iron oxides composite (Fe_3_O_4_/GO) for the pre-concentration and solidification of U(vi) ions from aqueous solutions.^79^ The U(vi) adsorption on Fe_3_O_4_/GO was strongly influenced by pH and was insensitive to ionic strength variation. The maximum U(vi) adsorption capacity was 69.49 mg g^−1^ at pH 5.5 in 0.01 M KNO_3_ solution and the adsorption was favored at elevated temperatures. Although the composite could be reused for six cycles, it was unstable due to considerable leaching of iron below pH 8 which is undesirable.

By integrating the multiple benefits of GO and magnetic PB nanoparticles, PB/Fe_3_O_4_/GO nanocomposite was prepared for the removal of radioactive cesium in water.^80^ Around 70% of Cs was adsorbed onto PB/Fe_3_O_4_/GO within 30 min and the maximum Cs uptake capacity was 55.56 mg g^−1^ in DI water. The enhanced adsorption efficiency and capacity was due to the anchoring technology, which reduced the aggregation of nanoparticles and increased the effective adsorption surface of the adsorbent. The composite was capable of removing Cs from real environmental samples and stable, but not readily regenerated.

Owing to high reactivity of amidoxime towards uranyl, amidoximated magnetite/graphene oxide (AOMGO) composite was prepared in two steps.^81^ The first step involved the simple co-precipitation of magnetic graphene oxide and the second step involved amidation of GO with diaminomaleonitrile followed by the treatment with hydroxylamine hydrochloride to obtain the final products. The resulting AOMGO composite showed maximum U(vi) adsorption capacity of 284.9 mg g^−1^ at pH 5 in 0.01 M NaClO_4_ solution which is relatively high due to formation of stable complexes between amidoxime and other oxygen-containing functional groups on the surfaces of AOMGO with U(vi).

Lately, CB[6]/GO/Fe_3_O_4_ was fabricated by linking the CB[6] *via* hydrogen bonding and introducing numerous well dispersed Fe_3_O_4_ nanoparticles by co-precipitation approach.^82^ The resultant composite was investigated for U(vi) removal and manifested the competitive adsorption performance and acceptable reusability. In addition, the composite could be regenerated by acidic solution in a contact time of 24 h, with only minute iron leaching.

#### 3.2.2 Inorganic clay

Clay minerals have a distinct layered morphology composed of the octahedral (Al^3+^, Fe^2+^, Fe^3+^, or Mg^2+^) and tetrahedral (Si^4+^) structures depending on the type of clay.^83^ Owing to isomorphic substitution, the clay minerals have surface charge which play an important role to capture metal ions. The use of clay minerals as substrate materials prevents the particle aggregation, enhances the dispersion as well as improves stability. Common clay minerals include kaolinite, bentonite, montmorillonite, layered double hydroxide, zeolite, attapulgite, sepiolite, halloysite, *etc.* At present, many kinds of clay minerals have been employed to prepare magnetic composites to adsorb contaminants from water.^84^

To investigate the adsorption behavior of U(vi) from aqueous solutions, β-cyclodextrin (β-CD) was chemically grafted onto halloysite nanotube/iron oxides and applied in batch system as a function of various environmental parameters.^85^ The maximum removal (∼92%) of initial 4.75 mg L^−1^ uranium was observed at pH 7 in 0.01 M NaNO_3_ solution. The pseudo-second-order model was found to be best correlated model with kinetic data, confirming that chemisorption was the rate-controlling mechanism. The removal efficacy of U(vi) on CD/HNT/iron oxide was higher than that on bare HNTs and HNT/iron oxides. This enhancement was caused by the multiple OH groups provided by surface-grafted β-CD. Satisfactory treatment efficiency was observed by CD/HNT/iron oxide in simulated wastewater. In addition, the CD/HNT/iron oxide was proposed as a backfilling material for a deep geological disposal of high-level radioactive waste.

The co-precipitation approach was adopted to synthesize attapulgite-iron oxide magnetic composites for the efficient pre-concentration of Eu(iii) from aqueous solution.^86^ The resultant Eu(iii) adsorption isotherms were well simulated by Langmuir model with and the maximum adsorption capacity of 117 mg g^−1^ at pH 5.0 in 0.01 M NaClO_4_ solution. The adsorption behavior was improved in the presence of humic acids and the observed enhancement in Eu(iii) adsorption at low pH was due to strong complexation of Eu(iii) with surface adsorbed humic acid on solid particles, whereas the reduction in adsorption at high pH was due to the formation of soluble Eu–HA complexes.

Very recently, Yang *et al.*^89^ reported the synthesis of chitosan grafted magnetic bentonite using a plasma induced grafting method and investigated its potential usage as an adsorbent for radioactive cesium in simulated groundwater and seawater (sampled from Pacific Ocean near Hamamatsu, Japan). The results showed enhanced coagulation due to plasma modification. In addition, this material exhibited good magnetic sensitivity as well as low turbidity and high stability in the seawater sample. Multifunctional adsorbent with superparamagnetic and PB particles was synthesized in the presence of sepiolite silicate (as porous platform) by one pot method.^87^ The efficacy of the resulting nanostructure was determined for the adsorption of Cs ions from aqueous media. The maximum Cs removal capacity was 102 mg g^−1^ at 22 °C in DI water. The thermodynamic studies suggested that Cs adsorption onto Sep/NPPB was a typical physio-sorption process due to free energy around −14.5 kJ mol^−1^.

Surface modification of a magnetic Mg–Al layered double hydroxide with citrate acid (C_6_H_5_O_7_^3−^) by ion exchange was carried out for the efficient removal of U(vi) that involved formation of the citrate–uranium complexes in the interlayer of the magnetic citrate Mg–Al layered double hydroxide.^88^ The adsorption process was in good agreement with Freundlich model and pseudo-second-order kinetics. The results demonstrated that the maximum adsorption capacity of 180 mg g^−1^ with the initial U(vi) concentration was 200 mg L^−1^ at 298 K in DI water.

## 4. Case studies

Although numerous studies have been conducted for the effective removal of radionuclides by magnetite-based adsorbents as presented in Tables 2 and 3. However, these studies present laboratory scale treatment. Therefore, the available information of pilot scale setup is still very scarce due to limited commercialization of operational setup and lack of regulatory framework for the use of magnetite adsorbents in water remediation. The representative case studies of magnetite-based adsorbents are summarized here to provide basic knowledge for applications to the real world environmental problems and to suggest the best option for their successful performance.

### 4.1 MAG*SEPSM technology for the decontamination of radioactive milk

**Fig. 5:**
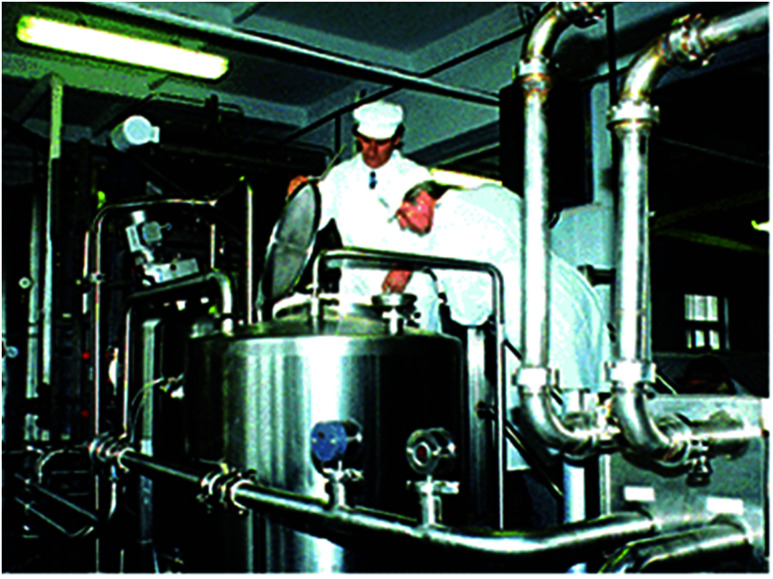
The MAG*SEP^SM^ milk decontamination system at Ovruch, Ukraine 1997.^1^

After the Chernobyl nuclear accident various radionuclides were expelled into the environment, including ^137^Cs which contaminated the grazing land for the cattle in the Ukraine. Consequently, milk products were contaminated and Cs levels were exceeding the international drinking standard of 370 Bq/L.^90^ To remove the radioactive cesium from milk, the MAG*SEP^SM^ treatment system was installed at the Ovruch Dairy in Ukraine (Fig. 5). It is a separation technology that uses magnetic particles to selectively adsorb contaminants such as heavy metals, radionuclides, or nitrates from contaminated water or other liquids. The vendor claimed that this technology could also treat the soil and could be operated *in situ* or *ex situ* to treat large volumes of liquids.

The MAG*SEP^SM^ particles have a magnetic core embedded in a polymer-based protective covering, and outer covering composed of a specific functional group. The demonstration tests were conducted at Argonne National Laboratory which revealed that the process could remove 95% of radioactive cesium from liquid milk. These particles had high adsorption capacity and good recyclability. However, these particles had a lack of selectivity and poor capturing ability when metals are present in various oxidation states. Low pH solutions significantly reduced the adsorption performance. In addition, the recycled MAG*SEP^SM^ particles contained the traces of contaminants that could not be used for different applications due to probable cause of cross contamination.

### 4.2 Large scale study using a drum magnetic type separator to decontaminate cesium eluted ash slurry

**Fig. 6:**
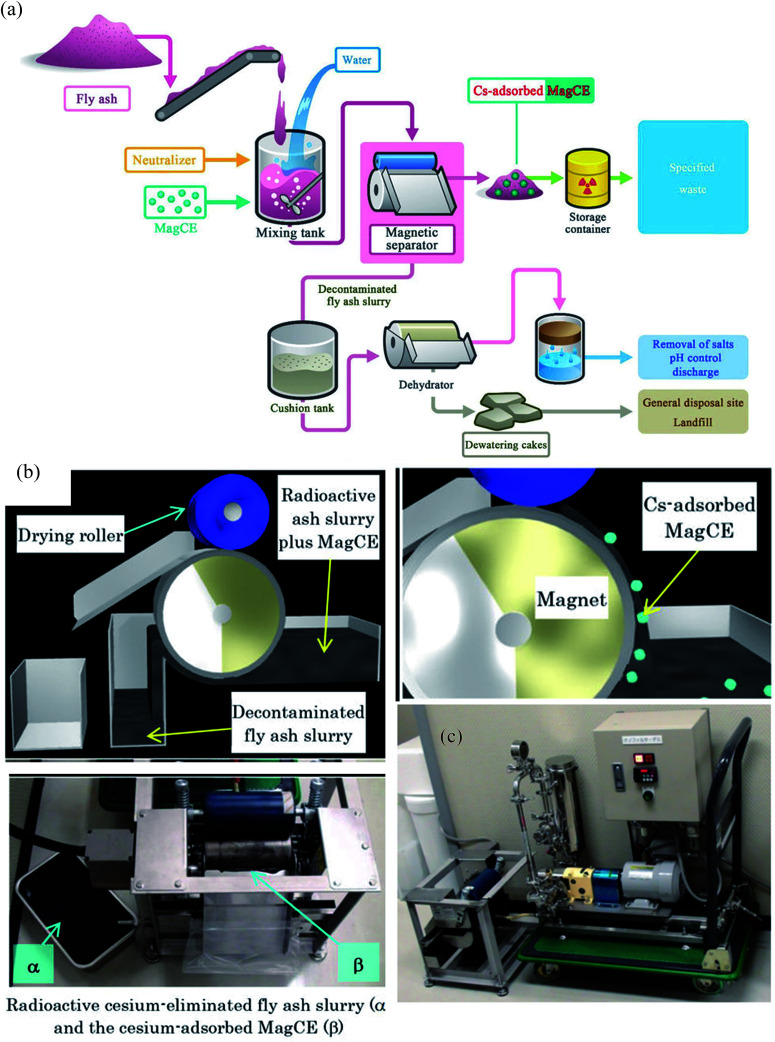
(a) Schematic representation of the concept of the compact decontamination system composed of a slurry mixing tank, a magnetic separator to recover the MagCE and the dewatering equipment. (b) Schematic representation of the decontamination involving removal of radioactive cesium using MagCE and a drum-type magnetic separator (c) pilot scale decontamination system.^91^

Around 140 000 tons of fly ash was generated by the Fukushima nuclear accident. This fly ash was concentrated with water-soluble radioactive cesium that could pose severe health issues by contaminating the groundwater. Conventional methods to remove Cs required a series of complicated steps including the separation of cesium eluted water from fly ash slurry, addition of flocculants and use of a decontaminant powder to bind cesium. However, the rapid separation of such a finely divided powder is problematic. In addition, cesium in dewatered fly ash cakes has a significant potential to leach and therefore, contaminating the natural resources. To decontaminate radioactive cesium, magnetically guidable cesium eliminator (MagCE) adsorbents were synthesized which have porous structure, ferromagnetic material, and alkaline-resistant nickel ferrocyanide.^91^ The direct addition of MagCE to the fly ash slurry and rapid magnetic separation reduced the number of steps for decontamination and could avoid cesium leaching from dewatered cakes. The large-scale (Fig. 6) demonstration experiments with fly ash (1 kg) in water (10 L) revealed >99% Cs removal from liquid component by MagCE using a drum type magnetic separator. The author proposed the use of MagCE for large scale removal of water soluble cesium and other radionuclides from soil and plant biomass.

## 5. Summary and recommendations

Traditional treatment technologies (coagulation, precipitation, ion exchange *etc.*) are energy-intensive and produce significant amounts of radioactive sludge. The use of nano-adsorbents in the decontamination of radioactive water is gaining interest by leaps and bounds. Especially the carbon based superparamagnetic composite adsorbents reduced solid waste production owing to their high removal capacities contributed by several factors (including exceptionally high surface area, magnetic susceptibility, and high chemical stability). In addition, the affectivity and selectivity of these adsorbents can be easily tuned by surface modification to capture low levels of radionuclides in bulk water. In contrast to conventional methods, less energy is required for the collection of magnetic particles by means of an external magnet.

So far, the behavior of magnetite-based adsorbents under irradiation has not been investigated therefore future research should be done to explore the radiations induced effect on magnetic adsorbents.

From the industrial point of view, it's necessary to focus on the simple, convenient and low-cost preparation methods avoiding harsh conditions. The precursors used for the synthesis and surface modification of the magnetic adsorbents should be ecologically benign. In addition, organic functionality should follow the CHON principle, *i.e.* (preferably composed of the elements C, H, O, and N), which implies that at the end of their useful life, they can be completely incinerated to minimize the generation of secondary waste. Ideally, the adsorbent should be chemically and radiolytically stable so that it can withstand the optimized conditions for several consecutive adsorptions and desorption cycles without leaching of the magnetic core, coating, and functional groups. High pre-concentration factor (which is the ratio of the solution volume before adsorption to the eluent volume during the desorption process) is desired to concentrate the recovered radionuclides from the bulk solution, so that recovered radionuclide could be safely disposed in a small volume. However, this factor has been overlooked and not discussed in details yet. Similarly, the distribution coefficient (which is important at dilute radionuclide concentrations) was not evaluated in detail in the reviewed literatures. Therefore, for practical perspectives, both these factors need to be considered for the future studies.

For large scale application of adsorbents short equilibration time is preferred for both adsorption and desorption processes to minimize the operation costs. Even though, the majority of documented studies focused on the small adsorption equilibrium time, desorption time was long (and/or not studied in detail) which might affect the economic feasibility of the process. Selectivity is a key parameter for any adsorbent because the presence of the common alkali and alkaline earth metal ions in most environmental water compete for the same binding sites, resulting in poor removal performance of the adsorbent towards the target radionuclide. The majority of the listed magnetic adsorbents do not exhibit high selectivity except for those incorporating certain functional groups *i.e.* Prussian blue, amido oxime, phosphonates *etc.*

The large number of reviewed adsorption studies were carried out in laboratory environments with controlled media and aqueous samples, not considering the complex environmental conditions of real contaminated sites. It is noteworthy to mention here that majority of these studies incorporate very high initial concentration of radionuclides which is not environmentally realistic.

The recent studies described the batch adsorption process in detail, which is a first stage to examine the efficacy of any adsorbent to remove radionuclides; nevertheless, it is not considered as a convenient approach for large scale water treatment. This issue could be compensated by the flow-through column based operation that could be used directly at a continuous water supply. However, many magnetic adsorbents have a powdered-like appearance, which could cause plugging issues and thus reduce flow rates and/or clog the filter pores. Fortunately, the synthesis of new porous magnetic composites seems to be a promising strategy to respond to the above mentioned technical problem. Moreover, its common practice to add organic binding agents as an additive in powdered adsorbent to make structured adsorbents (beads, granules or pellets) and to impart mechanical stability needed to withstand the stress during operation. However, the performance of the structured adsorbents was reduced due to pore blockage by the binder. In addition, there is possibility of leaching of binder which will in turn increase the Total Organic Carbon (TOC) of the treated water. Therefore, binder-free processing approaches such as pulsed current processing and/or 3D printing should be investigated for the bulk production of structured adsorbents.

Although, magnetite-based adsorbents have great advantages in comparison to conventional adsorbents but still their use is limited to labs scale water treatment due to their high production cost and the complex equipment required for the separation.

The continuous decline in adsorption performance was experienced from the recent pilot scale study due to the instability of magnetic adsorbent and unwanted accumulation of magnetic particles in the pipes and peripheral components resulting in the reduced flow rate.^143^ In addition, to separate the magnetic adsorbent, magnetic drum separator (MDS) and high gradient magnetic separator (HGMS) were used but MDS was unable to separate magnetic adsorbent completely while HGMS required regular flushing resulting in discontinuous operation and the dilution of the final particle concentrate. To avoid, aforementioned issues for the large-scale removal of radionuclides, it is suggested that one should use stable magnetic adsorbents and high-gradient filters (HGF) or a superconducting magnetic separation (SMS) system as a substitute to electromagnets. Finally, the cost of adsorbents should be carefully evaluated for their preparation and the regeneration for the long term industrial use.

Once these issues are properly addressed, these modified adsorbents with improved characteristics could be effectively utilized in closed system without clogging the filter pores, while in open environment these could be easily recovered and reused. Especially in the reactor decommissioning and/or nuclear accident situations, because robots could spread these adsorbents in high radiation fields and retrieve the captured radionuclides with help of high density electromagnet.

## Abbreviation

γ-MPS: γ-Methacryloxypropyltrimethoxysilane
AAM: Alginate agarose magnetite
AC: Activated carbon
AO: Amidoxime
AO/AMPS-MBA: Acrylamidoxime-*co*-2-acrylamido-2-methylpropane sulfonic acid crosslinked with *N*,*N*-methylenebisacrylamide
APS: Aminopropylsilane
APTMS (APMS): 3-Aminopropyl trimethoxysilane
BP: Tetraethyl-3-aminopropane-1,1-bisphosphonate
BMSPN: *N*,*N*′-Bis(3-methoxylsalicylidene)-1,2-phenylenediamine
CB[6]: Cucurbit[6]uril
CB-MNs: Calixarene-based magnetic nanoparticles
CMK-3: Mesoporous carbon
CMLH: Calcined magnetic layered double hydroxide/hydroxyapatite
CNTs: Carbon nanotubes
D2EHPA: Di-(2-ethylhexyl) phosphoric acid
DA: Dopamine
DPAO: Diacrylamidoxime triaethylenetetralevopimaramide
DPTS: Diethylphosphatoethyltriethoxysilane
D*t*BuCH18C6: Di-*tert*-butyl cyclohexano-18-crown-6
DTC: Dithiocarbamate
EDA-MCCS: Ethylene diamine magnetic carboxymethyl chitosan nanoparticles
EMMC: Ethylenediamine-modified magnetic chitosan
GMA/BPA: Glycidyl methacrylate/*N*,*N*′-methylenebisacrylamide
GO: Graphene oxide
HA: Humic acid
HCC: Hydrothermal cross-linking chitosan
HNT: Halloysite nanotube
IIP: Ion imprinted polymer
IMCR: Ion-imprinted magnetic chitosan resins
KTiFC: Potassium titanium ferrocyanide
LDH: Layered double hydroxide
MMSNs–PP: Magnetic mesoporous silica nanoparticles–phosphonate
MMT: Montmorillonite
MNC: Magnetic nanocluster
MS: Magnesium silicate
MZC: Magnetic zeolite composite
PAM: Polyacrylamide
PA-SMM: Phosphonic acid-functionalized silica magnetic adsorbent
PB: Prussian blue
P(GMA–AA–MMA): Poly glycidyl methacrylate acrylic acid methyl methacrylate
PAMAMG3: Third generation poly(amido) amine
PVA: Polyvinyl alcohol
SDS: Sodium dodecyl sulfate
TETA-MCS: Triethylene-tetramine modified magnetic chitosan adsorbents
TMS: *N*-[(3-Trimethoxysilyl)propyl]ethylenediamine triaceticacid trisodium

## Conflicts of interest

There are no conflicts to declare.

## Supplementary Material
